# Baseline predictors of antiretroviral treatment failure and lost to follow up in a multicenter countrywide HIV-1 cohort study in Ethiopia

**DOI:** 10.1371/journal.pone.0200505

**Published:** 2018-07-11

**Authors:** Nigus Fikrie Telele, Amare Worku Kalu, Gaetano Marrone, Solomon Gebre-Selassie, Daniel Fekade, Belete Tegbaru, Anders Sönnerborg

**Affiliations:** 1 Division of Clinical Microbiology, Department of Laboratory Medicine, Karolinska Institute, Karolinska University Hospital, Stockholm, Sweden; 2 Department of Microbiology, Immunology and Parasitology, Addis Ababa University, Addis Ababa, Ethiopia; 3 Division of Infectious Diseases, Department of Medicine Huddinge, Karolinska Institute, Karolinska University Hospital, Stockholm, Sweden; 4 Department of Infectious Diseases, Addis Ababa University, Addis Ababa, Ethiopia; 5 Ethiopian Public Health Institute, Addis Ababa, Ethiopia; University of Massachusetts Medical School, UNITED STATES

## Abstract

**Background:**

Antiretroviral therapy (ART) has been rapidly scaled up in Ethiopia since 2005, but factors influencing the outcome are poorly studied. We therefore analysed baseline predictors of first-line ART outcome after 6 and 12 months.

**Material and methods:**

874 HIV-infected patients, who started first-line ART, were enrolled in a countrywide prospective cohort. Two outcomes were defined: i) treatment failure: detectable viremia or lost-to-follow-up (LTFU) (confirmed death, moved from study sites or similar reasons); ii) LTFU only. Using stepwise logistic regression, four multivariable models identified baseline predictors for odds of treatment failure and LTFU.

**Results:**

The treatment failure rates were 23.3% and 33.9% at 6 and 12 months, respectively. Opportunistic infections (OI), tuberculosis (TB), CD4 cells <50/μl, and viral load >5 log10 copies/ml increased the odds of treatment failure both at 6 and 12 months. The odds of LTFU at month 6 increased with baseline functional disabilities, WHO stage III/IV, and CD4 cells <50/μl. TB also increased the odds at month 12. Importantly, ART outcome differed across hospitals. Compared to the national hospital in Addis Ababa, patients from most regional sites had higher odds of treatment failure and/or LTFU at month 6 and/or 12, with the exception of one clinic (Jimma), which had lower odds of failure at month 6.

**Conclusions:**

In this first countrywide Ethiopian HIV cohort, a high ART failure rate was identified, to the largest extent due to LTFU, including death. The geographical region where the patients were treated was a strong baseline predictor of ART failure. The difference in ART outcome across hospitals calls the need for provision of more national support at regional level.

## Introduction

The incidence and prevalence of the human immunodeficiency virus type 1 (HIV-1) infection in Ethiopia has decreased over the years [1], but still 730 000 people are estimated living with virus in 2018, corresponding to an adult prevalence of 1.15% [2]. However, marked variations between urban and rural areas as well as across regions reported; urban areas showing a seven-fold higher prevalence [3]. Ethiopia has adapted the WHO clinical staging system in order to include a list of the common infections and diseases occurring among PLHIV in an Ethiopian context [2]. This is used to identify patients who need urgent clinical assessment and referral to the nearest health facility to receive standardised care and treatment interventions. The four WHO clinical stages, which have been established for persons with confirmed HIV infection include: stage I- no symptoms; stage II- mild symptoms; stage III- advanced symptoms; and stage IV- severe symptoms [4]. The WHO clinical staging differentiates pulmonary TB (stage III) from extrapulmonary TB (stage IV or equivalent of AIDS).

Since 2005, antiretroviral therapy (ART) has been made widely accessible in Ethiopia and there were 420 000 people living with HIV (PLHIV) on ART with 59% coverage by 2016 [5]. However, more than one-fourth of those who started ART have dropped out from the health care follow-up [1]. Thus, discontinuation of ART and lost-to-follow-up (LTFU) are key problems for the health care of PLHIV. For Ethiopia, large differences in the proportion of LTFLU have been reported, ranging from 9.8% up to 31.4% in different regions of the country [6]. The risk factors for discontinuation are still poorly understood in many low-income countries (LIC), including Ethiopia.

In LIC, viral load (VL) has seldom been used for monitoring of ART, due to economic and logistic reasons. Hence, despite the WHO recommendation from 2013 to use VL [7], most sub Saharan Africa countries (sSA) still use only clinical and immunologic criteria, including Ethiopia. In the present study, VL was analysed retrospectively for scientific purposes, but the data was not used in the clinical care since the tests were done in batch years after study initiation.

Despite a large number of patients receiving ART in Ethiopia, there are few reports on ART outcome, from only a few regions of the country. In order to fill this knowledge gap, this study aimed to identify baseline predictors of first-line ART outcome among PLHIV. Thus, unlike previous studies, this study has a wide geographical coverage, providing a more complete picture on the ART outcome at the national level.

## Material and methods

### Study design and study population

The study was conducted on PLHIV, who were enrolled in the Advanced Clinical Monitoring of Antiretroviral Treatment in Ethiopia (ACM) cohort [8,9]. Between October 2009 and December 2011, 874 ART naïve HIV-1 infected patients (females: 527; males: 347; age: ≥14 years) were enrolled at seven university hospitals (range per site: 112 to 134) and started ART as per the national guideline [10] with a follow-up until 2013 (median follow up time: 2.1 years; IQR: 1.4 to 2.9). As per the national guidelines, patients were given the following fixed dose combinations (FDC) first-line ART regimens: TDF+3TC+EFV; TDF+3TC+NVP; ZDV+3TC+EFV, ZDV+3TC+EFV; d4T+3TC+EFV; or d4T+3TC+NVP. The recommended doses of the drugs include TDF 300 mg once daily; ZDV: 300 mg every 12 hours; d4T: 30 mg every 12 hours; 3TC: 300 mg once daily or 150 mg every 12 hours; NVP: 200 mg daily for the first 2 weeks, followed by 200 mg every 12 hours; and EFV: 600 mg once daily [10].

The hospitals were distributed all over Ethiopia: Tikur Anbessa Specialized Hospital (TASH), a tertiary level national hospital in the capital Addis Ababa- Central region; Gondar- Northwest; Jimma- West; Mekelle- North; Harrar- East; Hawassa- South; and a mobile army unit at the Armed Forces General Hospital.

### Data and specimens

Clinical assessment and routine laboratory tests were performed at the study sites, using national ART clinic forms and hospitalization/intervening care forms. Data were entered into a site database and later uploaded to a central database at the Ethiopian Health and Nutrition Research Institute (EHNRI) after quality control checks. For this study, the following data were extracted at baseline, month 6 and 12 of ART from the central database: WHO clinical stage, onset or relapse of opportunistic infections (OI), weight, body mass index (BMI), ART regimen, months on ART, CD4 and CD8 cell counts/percentages, and plasma HIV-1 RNA load (VL). Tuberculosis (TB) was considered if an active TB was diagnosed or if the patient was receiving anti-TB therapy at ART initiation. CD4 and CD8 cells were determined at the local laboratories using BD FACSCalibur machines (Becton Dickinson, San Jose, USA). VL was analysed centrally at EHNRI by NucliSENS easyQ^®^ HIV-1 Nucleic Acid Sequence-Based-Amplification (NASBA) assay (BioMérieux Diagnostics) with a lower detection limit of 150 HIV-1 RNA copies/ml.

### Outcome measures and statistical analyses

Two ART outcome measures were defined at month 6 and 12: i) treatment failure defined as either failure to attain viral suppression (virologic failure) or LTFU (confirmed death, moved from study sites, transferred to other health facilities, refused further contact or similar reasons); ii) LTFU only. Both on-treatment and intention-to-treat (ITT) analysis were employed to assess ART outcome. Treatment failure at month 6 and 12 was defined using two threshold VL: 150 copies/ml (limit of detection of the assay) and 1000 copies/ml (WHO definition) [11].

Binary logistic regression models were used to identify significant baseline predictors of treatment failure and LTFU at month 6 and 12. The following variables were considered as potential baseline predictors: study site, gender, age, educational status, employment status, marital status, spouse HIV serostatus, functional status, WHO clinical stage, TB, OI, treatment regimen (NRTI or NNRTI), BMI, weight, CD4 cells, CD4/CD8 ratio, and VL.

Baseline factors that predicted the ART outcome in the bivariate analyses at 20% significance level were included in the final multivariable analysis for treatment failure and LTFU. At the beginning, six multivariable models were developed: four to assess treatment failures after month 6 and 12 (using two VL threshold values: 150 and 1000 copies/ml), and two for LTFU at month 6 and 12. There were no substantial differences between the models developed to assess the treatment outcomes using the two VL threshold values (data not shown) and hence we used the 1000 copies/ml threshold and remained with two models predicting the treatment outcomes (at month 6 and 12). Finally, a total of four predictive models were presented to assess predictors of treatment failure outcomes and LTFU at month 6 and 12.

Both stepwise forward and backward methods in multivariable logistic regression at *p* = 0.1 significance level were used to select variables included in the best final models. Odds ratios (OR) with the 95% confidence intervals were presented. P-value <0.05 was considered significant in the final multivariable models. Data analysis was performed using STATA software 14 (Stata Corp. College Station, Texas, USA).

### Ethical considerations

The study was approved by the National Research Ethics Review Committee in Ethiopia, and Institutional Review Board (IRB) of EHNRI. Written informed consent was obtained from all participants.

## Results

### Baseline characteristics

A description of the baseline socio-demographic, clinical, and biomedical characteristics is given in Table 1 and Table 2. In brief, the majority (60%, 527/874) of the participants were females, who were younger than males (mean ± SD age: 32.4 ± 8.4 years vs 37.1 ±8.7 years; p<0.001). Males had more often advanced WHO stages (III or IV) than females (63.7% vs 51.4%, p<0.001). Most participants (79%) were working, 18% ambulatory and 3% bedridden. Site-specific differences were observed both for functional status (p<0.001) and for clinical stages (p<0.001), where 66%, 61%, and 58% of the patients from Army, TASH and Hawassa, respectively, had WHO stage III or IV. The mean ± SD BMI of the patients was 19.4 ± 3.1 kg/m^2^ of whom 41.5% were underweight (<18.5 kg/m^2^).

**Table 1:** Sociodemographic characteristics of 874 HIV-infected patients at antiretroviral therapy initiation.

Variables	Categories	Frequency n (%)
**Gender**	Female	527 (60.3)
	Male	347 (39.7)
**Age (years)**	14–24	77 (8.8)
	25–34	393 (45.0)
	35–44	282 (32.3)
	45–54	98 (11.2)
	≥55	24 (2.7)
**Marital status**	Never Married	156 (18.0)
	Married	428 (49.3)
	Divorced	178 (20.5)
	Widow(er)	106 (12.2)
**Partners HIV status**	Positive	207 (64.9)
	Negative	112 (34.1)
**Educational status**	No education	155 (18.0)
	Primary	313 (36.5)
	Secondary	283 (33.0)
	Tertiary	107 (12.5)
**Occupation**	Unemployed	400 (48.0)
	Specified job	193 (23.2)
	Employed	240 (28.8)

**Table 2:** Clinical and laboratory parameters of 874 HIV-infected patients at antiretroviral therapy initiation.

Variables	Categories	Frequency n (%)
**Functional status**	Bedridden	25 (2.9)
	Ambulatory	155 (17.8)
	Working	691 (79.3)
**WHO clinical stage**	I	149 (17.0)
	II	223 (25.5)
	III	372 (42.6)
	IV	130 (14.9)
**TB screen**	Positive	198 (24.0)
	Negative	626 (76.0)
**OIs**	≥1 OI	334 (38.4)
	None	355 (61.6)
**NRTI regimen**	d4T-based	44 (5.0)
	ZDV-based	362 (41.4)
	TDF-based	467 (53.4)
**NNRTI regimen**	NVP	380 (43.5)
	EFV	494 (56.5)
**BMI (mean** ± **SD kg/m**^**2**^**)**		19.4 ± 3.1
**Weight (mean** ± **SD kg)**		52 ± 10
**CD4 count (mean** ± **SD cells/μl)**		143.8 ± 87
**Viral load (mean** ± **SD log10 copies/ml)**		5.2 ± 0.8

NRTI: nucleoside-analogue reverse transcriptase inhibitors; d4T: stavudine; ZDV: zidovudine: TDF: tenofovir; NNRTI: non-nucleoside-analogue reverse transcriptase inhibitors; NVP: nevirapine; EFV: efavirenz; OI: opportunistic infections.

One-fourth of the participants (males 34%, 108/320; females 18%, 90/504; p<0.0001) with clinical data (n = 824) were TB co-infected: 64% pulmonary TB (PTB); 30% extra-pulmonary TB (EPTB); and 6% unknown type. TB patients had lower CD4 counts (mean ± SD: 121 ± 86 cells/μl vs 154 ± 89 cells/μl; p<0.0001), and higher VL (mean ± SD: 5.4 ± 0.8 vs 5.1 ± 0.8 log10 copies/ml; p<0.0001) than TB negative patients. Among 869 patients with clinical data, one or more other OI and/or AIDS-defining conditions were found in 38.4% (males 43.6% vs females 35.0%; p<0.05).

The mean ± SD CD4 count (n = 870) was 144 ± cells/μl (75% had <200 cells/μl; 55% <150; 36% <100; and 14% <50. Males (n = 346) had lower CD4 count than females (n = 524) (mean ± SD: 132 ± 97 cells/μl vs 151 ± 80 cells/μl; p<0.01). Plasma HIV-1 RNA was detected in 99% (n = 844/855) subjects (mean ± SD VL: 5.2 ± 0.8 log_10_ copies/ml).

## Treatment regimens

Among 837 participants with data about criteria for ART initiation, 590 (70.5%) started ART based on CD4 count; 230 (27.5%) on clinical presentations plus CD4 count; and 17 (2%) on clinical presentation only. All participants were prescribed two NRTI (tenofovir (TDF): 53.4%; zidovudine (ZDV): 41.4%; stavudine (d4T): 5.0%; abacavir (ABC): 0.2%; all with 3TC) and one NNRTI (efavirenz (EFV): 56.5%; nevirapine (NVP): 43.5%).

### Treatment outcome at 6 and 12 months

**Fig 1:**
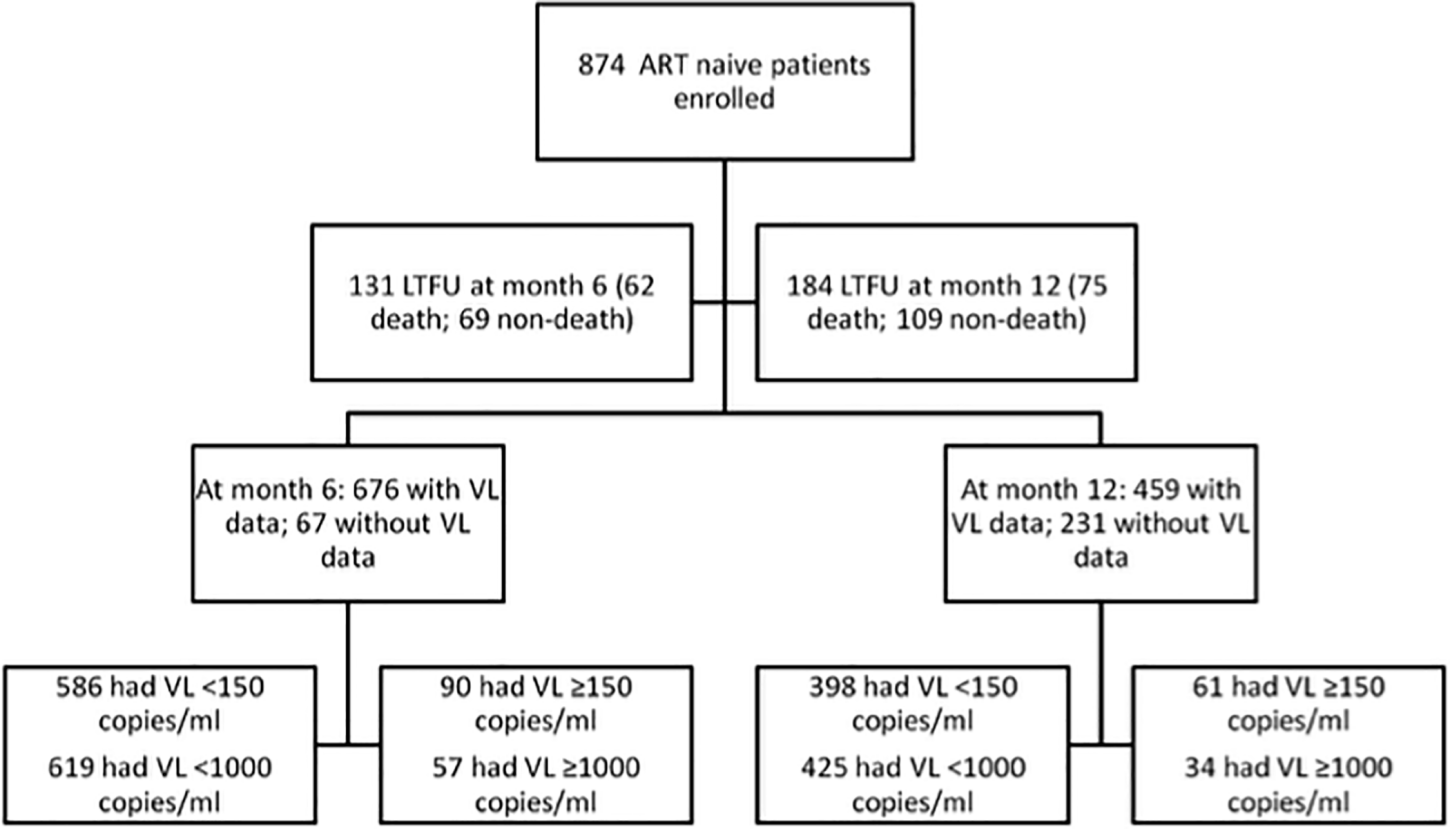
Profile of the study cohort during the first year of treatment follow up. ART = antiretroviral therapy; LTFU = lost-to-follow-up; VL = viral load.

At month 6, 131/874 (15.0%) patients were dead (n = 62) or LTFU due to other reasons (n = 69). A further 67 (8%) participants were still retained, but were excluded from the analysis due to lack of VL (Fig 1). Of the remaining 676 subjects, 90 (13.3%) had >150 copies/ml; 26 (3.8%) had 150–399; 7 (1.0%) had 400–999; and 57 (8.4%) had ≥1000. Hence, in the ITT analysis, the treatment failure rate was 27.3%. When virologic failure was redefined as VL ≥1000 copies/ml, the treatment failure rate was 23.3%.

At month 12, 184/874 (21.1%) were dead (n = 75) or LTFU due to other reasons (n = 109). A further 231 subjects were still retained, but were excluded from the analysis due to lack of VL (Fig 1). There was no significant difference between patients with or without VL at month 6 and/or 12 in terms of gender, age, functional status, WHO clinical stages, CD4 count and VL at baseline. Of the 459 remaining patients, 61 (13.3%) had >150 copies/ml; 17 (3.7%) had 150–399; 10(2.2%) had 400–999; and 34 (7.4%) had >1000. Detectable viremia was found in 28 of the 61 (46%) patients at both time points. In the ITT analysis, rates of treatment failure were 38.1% or 33.9%, respectively, when 150 or 1000 copies/ml threshold were considered.

### Baseline predictors of treatment failure

At bivariate analyses study site, gender, marital status, baseline functional status, WHO stage, TB, OI, CD4 count, and VL were significantly associated with treatment failure at month 6 and 12 (except for marital status, and WHO stage (p = 0.056).

At month 6, in the multivariable model gender, TB, OI, CD4 count, VL and study sites predicted the odds of treatment failure (Table 3). Males had higher odds (OR: 1.56; 95% CI: 1.14 to 2.14) than females. TB patients had higher odds (OR: 1.75; 95% CI: 1.23 to 2.50) than those without. Participants with OI had higher odds (OR: 1.44; 95% CI: 1.05 to 1.97) than those without. The odds was triple for patients who started ART with CD4 count <50 cells/μl (OR: 3.0; 95% CI: 1.77 to 5.07) compared to those with >200 cells/μl. Patients who had >5 log10 RNA copies/ml had nearly double odds of failure (OR: 1.98; 95% CI: 0.94 to 4.19) compared to those with <4 log10 copies/ml. Patients from Jimma had reduced odds (OR: 0.48; 95% CI: 0.25 to 0.93) compared to those from TASH, including a low failure rate (14%).

**Table 3:** Multivariable associations between baseline characteristics and treatment failure at 6 and 12 months.

Multivariable			
Predictive factors	Categories	Month 6 OR (95% CI)	Month 12 OR (95% CI)
**Hospital**	TASH	1	1
	Army	NS	NS
	Gondar	NS	NS
	Jimma	0.48 (0.25; 0.93)	NS
	Mekelle	NS	2.01 (1.11; 3.64)
	Harrar	NS	3.89 (2.04; 7.39)
	Hawassa	NS	2.75 (1.53; 4.94)
**Gender**	Female	1	
	Male	1.56 (1.14; 2.14)	
**Functional status**	Working		1
	Ambulatory		2.06 (1.38; 3.06)
	Bedridden		2.32 (0.94; 5.71)
**TB**	Negative	1	1
	Positive	1.75 (1.23; 2.50)	1.81 (1.25; 2.61)
**OI**	None	1	
	≥1 OI	1.44 (1.05; 1.97)	
**CD4 (cells/μl)**	>200	1	1
	150–200	NS	NS
	100–149	NS	NS
	50–99	NS	1.67 (1.01; 2.76)
	<50	3.00 (1.77; 5.07)	2.54 (1.49; 4.30)
**VL (copies/ml)**	<4 log10	1	
	4 log10–5 log10	NS	
	>5 log10	1.98 (0.94; 4.19)	

NS = not significant; OR = odds ratio; CI = confidence interval; TB = tuberculosis; OI = opportunistic infection; VL = viral load; stepwise multiple regression used to develop the methods.

At month 12, functional status, TB, CD4 count and study site, predicted treatment failure (Table 3). The odds was more than twice higher for participants who started ART with ambulatory (OR: 2.06; 95% CI: 1.38 to 3.06) and bedridden functional status (OR: 2.32; 95% CI: 0.94 to 5.71) as compared to those with working status. TB patients had higher odds (OR: 1.81; 95% CI: 1.25 to 2.61) than those without. Participants with CD4 count < 100 cells/μl had higher odds (OR: 1.67; 95% CI: 1.01 to 2.76 for 99–50 cells/μl; OR: 2.54; 95% CI: 1.49 to 4.30 for <50 CD4 cells/μl) as compared to those with >200 cells/μl.

**Fig 2:**
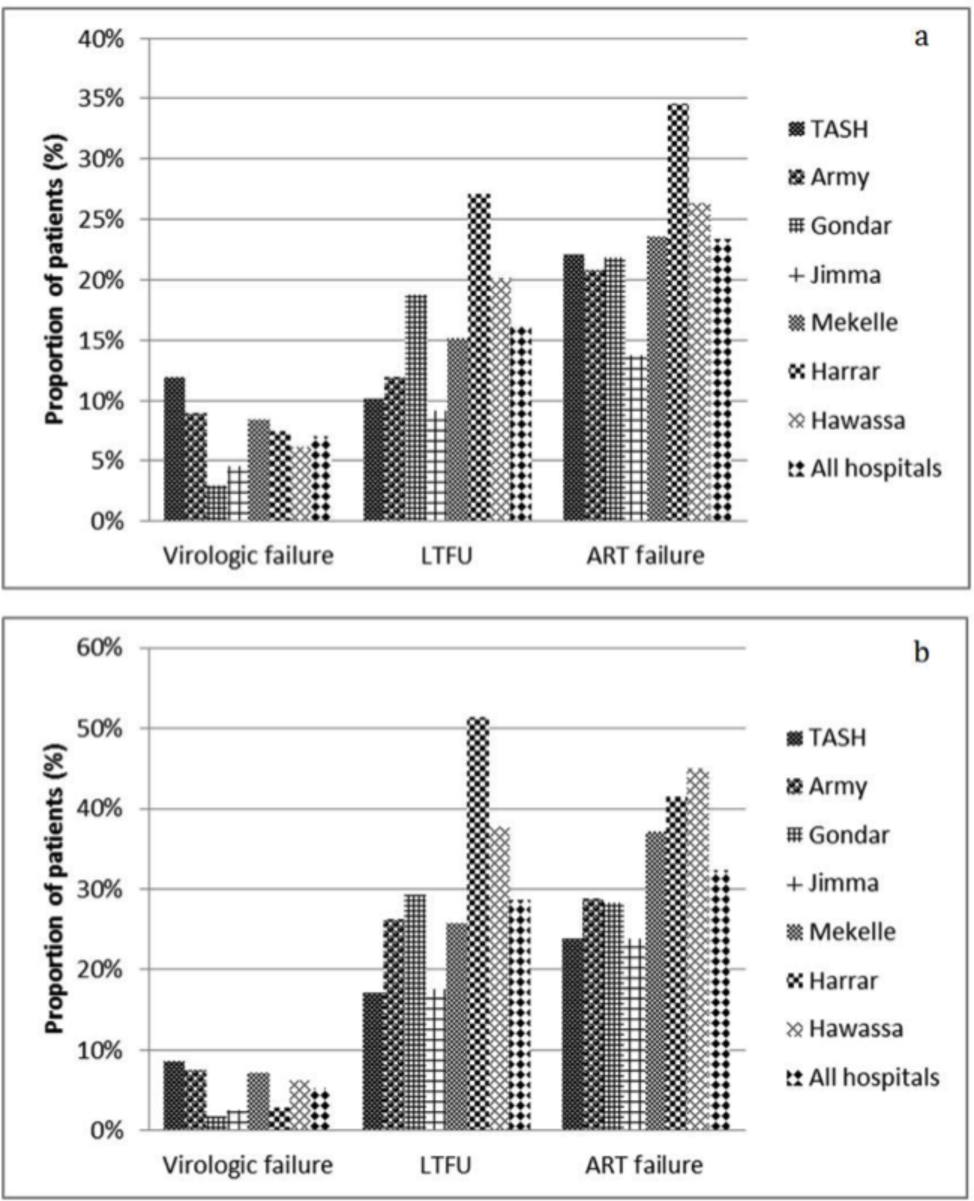
Antiretroviral treatment (ART) outcome at month six (a) and 12 (b) for 874 patients. The number of patients with treatment outcome data at month six and 12 were: Tikur Anbessa Specialized Hospital (TASH): 109, 105; Army: 101, 80; Gondar: 133, 113; Jimma: 109, 80; Mekelle: 119, 97; Harrar: 107, 70; Hawassa: 129, 98. Virologic failure = VL> 1000 copies/ml; LTFU = lost-to-follow-up due to death and other reasons; ART failure = virologic failure plus LTFU.

The study site differences were large. Patients from the regional hospitals Harrar, Hawassa, and Mekelle had nearly quadruple, triple and double odds of treatment failure (OR: 3.89; 95% CI: 2.04 to 7.39; OR: 2.75; 95% CI: 1.53 to 4.94; OR: 2.01; 95% CI: 1.11 to 3.64) as compared to those from the national tertiary level hospital TASH (Table 3). The treatment failure rate was thus significantly higher among patients from Harrar (41%), Hawassa (45%) and Mekelle (37%) compared to TASH (24%) (Fig 2).

### Baseline factors associated with LTFU

Bivariate analyses revealed that study site, gender, functional status, WHO stage, TB, CD4 cells, and VL were significantly associated with odds of LTFU, including deaths, at month 6 and 12. Also, OI was significantly associated at month 12. We excluded BMI and CD4:CD8 ratios because of high number of missing values.

At month 6, the multivariable model revealed functional status, WHO stage, CD4 cells and study sites as predictors of LTFU (Table 4). Ambulatory and bedridden patients had double odds (OR: 2.37; 95% CI: 1.54 to 3.63; OR: 2.22; 95% CI: 0.86 to 5.71) as compared to those who were working. Patients ith WHO stage III/IV had higher odds (OR: 1.79; 95% CI: 1.21 to 2.37) compared to those with WHO stage I/II. The odds of LTFU was double for patients who started ART with CD4 count < 50 cells/μl (OR: 2.32; 95% CI: 1.28 to 4.19) compared to those with >200 cells/μl.

**Table 4:** Multivariable associations between baseline characteristics and lost to follow up, including death, during 6 and 12 months of treatment.

		Multivariable	
Predictive factors	Categories	Month 6	Month 12
		OR (95% CI)	OR (95% CI)
**Hospital**	TASH	1	1
	Army	NS	NS
	Gondar	2.25 (1.06; 4.80)	1.83 (0.97; 3.47)
	Jimma	NS	NS
	Mekelle	NS	NS
	Harrar	2.75 (1.30; 5.81)	2.20 (1.17; 3.13)
	Hawassa	2.39 (1.12; 5.07)	2.16 (1.15; 4.06)
**Gender**	Female		1
	Male		1.71 (1.23; 2.37)
**Functional status**	Working	1	1
	Ambulatory	2.37 (1.54; 3.63)	2.20 (1.49; 3.24)
	Bedridden	2.22 (0.86; 5.71)	3.63 (1.61; 8.18)
**WHO clinical stage**	I/II	1	1
	III/IV	1.79 (1.21; 2.65)	1.92 (1.36; 2.72)
**TB**	Negative		1
	Positive		1.97 (1.37; 2.84)
**CD4 (cells/μl)**	>200	1	1
	150–200	NS	NS
	100–149	NS	NS
	50–99	NS	1.56 (0.95; 2.55)
	<50	2.32 (1.28; 4.19)	2.65 (1.57; 4.46)

NS = not significant; OR = odds ratio; CI = confidence interval; TB = tuberculosis; stepwise multiple regression used to develop the methods.

Patients from Harrar, Hawassa and Gondar had higher odds (OR: 2.75; 95% CI: 1.30 to 5.81; OR: 2.39; 95% CI: 1.12 to 5.07; OR: 2.25; 95% CI: 1.06 to 4.80, respectively) as compared to those from TASH. Compared to TASH (10%), patients from Harrar (27%), Hawassa (20%), and Gondar (19%) had higher LTFU rates of both at month 6 and 12 (Fig 2).

At month 12, gender, functional status, WHO stage, TB, CD4 cell count and study site, predicted the odds of LTFU (Table 4). Males had higher odds (OR: 1.71; 95% CI: 1.23 to 2.37) than females. Participants who were ambulatory or bedridden had more than double and triple odds (OR: 2.20; 95% CI: 1.49 to 3.24; OR: 3.63; 95% CI: 1.61 to 8.18) compared to those working. WHO stage III/IV patients had nearly double odds (OR: 1.92; 95% CI: 1.36 to 2.72) compared to those who had WHO stage I/II. TB patients had double odds (OR: 1.97; 95% CI: 1.37 to 2.84) compared to TB negatives. The odds for patients with CD4 cells/μl of 99–50 or <50 were higher (OR: 1.56; 95% CI: 0.95 to 2.55; OR: 2.65; 95% CI: 1.57 to 4.46) compared to those with >200 cells/μl.

Participants from Harrar, Hawassa, and Gondar had double odds (OR: 2.20; 95% CI: 1.17 to 3.13; OR: 2.16; 95% CI: 1.15 to 4.06; OR: 1.83; 95% CI: 0.97 to 3.47) compared to those from TASH. The LTFU rates were thus significantly higher in the regional hospitals (Harrar: 51%; Hawassa: 38%; Gondar: 29%) compared to TASH (17%) (Fig 2).

## Discussion

This is the first countrywide prospective longitudinal study on ART outcome in Ethiopia. Using the WHO definition and OT analysis, the virological failure rate was 8.4% at month 6 and 7.4% at month 12. In the ITT analysis, the treatment failure rates, including both virological failure and LTFU due to death or other reasons, were much higher, 23.3% and 33.9%. The baseline factors associated with increased odds of treatment failure and LTFU were in line with those reported from other sSA countries. However, the countrywide design of our study allowed us to also identify the study site as a strong predictor for treatment failure and LTFU.

The LTFU rate (including deaths) at month 6 (15%) and 12 (21%) were consistent with previous studies from other sSA countries, confirming that early death and LTFU are key features also in Ethiopia [12]. The LTFU rates of our nationwide study are in line with earlier smaller and regional studies from Bench Maji, Jimma and Gondar, which reported rates of 26.7%, 28%, and 31.4%, respectively [6]. As expected, the death rate was highest during the first 6 months. In contrast, the LTFU rates due to other reasons than death were similar during the first and second 6 month periods. Thus, retention in care is critical for the long-term outcome of ART, both continued medical and supportive efforts are needed to reduce mortality and LTFU.

In the OT analysis, using >1000 copies/ml cut-off, the virologic failure at month 6 and 12 (8.4% and 7.4%) were comparable to most other sSA studies [13–16] as well as to a limited study from Jimma, Ethiopia [17]. In our study, the national immunoclinical failure criteria dictated when to switch ART and the VL test was done retrospectively. Almost half of the patients who retrospectively were shown to have detectable viremia at month 6 continued their initial treatment with an ongoing viral failure up to month 12. This confirms the lack of precision of identifying ART failure using immunoclinical criteria and thereby the increased risk of developing an advanced drug resistance [18].

Overall the ITT analysis showed a high rate of treatment failure; 23.3% and 33.9% at 6 and 12 months, respectively. One possible reason for the observed high rate of treatment failure is the late presentation of patients as the majority of our study participants had low CD4 cell counts at ART initiation. Our study thus showed that despite better availability of ART in low and middle income countries, yet a substantial number of people are diagnosed/present late and start ART at later stages of the disease as described elsewhere [19,20]. In addition, the deficiencies in the health care system and the large number of PLHIV accessing ART in the country are outstanding challenges to provide all the necessary and standard monitoring strategies, which could ultimately contributed to the observed high rate of treatment failure. This is possibly reflected by the observed differences in treatment outcome between the national tertiary hospital and the regional hospitals from which patients were recruited for this study.

In Ethiopia, even though it has been reported that a large number of HIV-infected patients discontinue their treatment, so far only little research including a limited number of patients has been published [6]. In our nationwide study, the baseline parameters predicting treatment failure including LTFU (gender, functional status, TB, OI, CD4 count, VL) were well in line with what has been shown in other sSA countries. E.g. males failed more often than females [21] [22], as well as patients who started ART with clinical or laboratory signs of advanced disease. Of special concern is that most parameters showed that the majority of patients had a very advanced HIV disease at diagnosis. E.g. the mean CD4 count was 144/μl and 36% of participants started ART with cells <100/μl. Also, one fourth had TB at inclusion, which is more than two-folds higher compared to a study from Addis Ababa [23]. Although it has been reported that EPTB is more frequent than PTB among HIV/TB co-infected patients [24], the majority of our TB positive patients had PTB. This could possibly be explained by the fact that our patients were categorized as PTB as per the WHO guidelines [25].

The advanced immunodeficiency at baseline in a high proportion of our patients calls for national strategies to overcome the problem, e.g. through the test and treat approach. In a study from Kenya and Uganda such approach was implemented which resulted in that at the population level the viral suppression target of UNAIDS was reached within two years [16]. In contrast, a lowering of HIV incidence was not found in a recent study from South Africa using universal test and treat, which was due to poor linkage to care [15]. Thus, a change to universal test and treat in Ethiopia without improving access to health care is unlikely to reduce HIV incidence.

The importance of the health care structure is illustrated by the intriguing and important finding in our study that the study sites were strong predictors of ART outcome. It is known that there are substantial regional variations in Ethiopia with regard to knowledge towards HIV and its prevention, as well as in prevalence of HIV and other sexually transmitted infections (STIs) [26,27]. Our study is the first to show that there are also significant differences in outcome of ART across regions in Ethiopia. These differences could possibly be explained by sociodemographic and sociocultural variations as well as cultural and religious practices in the different regions, as earlier reported for other STIs [26]. E.g. it has been reported that frequent use of khat is a predictor of LTFU in eastern Ethiopia (Dire Dawa and Harar) [28]. Such sociocultural barriers could also contribute to the late presentation of patients at the HIV clinics and late initiation of ART. It is also known that a shorter distance to care decreases the LTFU and that decentralization of HIV care therefore is important [29]. In the present study, all patients were followed at the national hospital in Addis Ababa or at regional hospitals, where a long distance to care for a substantial number of patients is not unlikely to have contributed to the high rate of LTFU.

In conclusion, in this first nationwide HIV cohort study in Ethiopia a high rate of treatment failure was identified. Early death dominated during the first 6 months and LTFU during the first year, rather than virological treatment failure among those retained in care. In addition to well established baseline predictors, such as advanced immunodeficiency, TB and OIs, we identified the care-giving hospital as a major predictive factor of ART failure. Provision of further national support at the regional levels in Ethiopia is therefore essential, both for an earlier initiation of ART [30] as well as for improved retention of patients in care.
